# Association Between Ghrelin and Body Weight Trajectory in Individuals With Anorexia Nervosa

**DOI:** 10.1001/jamanetworkopen.2023.4625

**Published:** 2023-03-24

**Authors:** Youngjung R. Kim, Meghan S. Lauze, Meghan Slattery, Roy H. Perlis, Laura M. Holsen, Lauren Breithaupt, Casey M. Stern, Maurizio Fava, Jennifer J. Thomas, Elizabeth A. Lawson, Madhusmita Misra, Kamryn T. Eddy

**Affiliations:** 1 Center for Quantitative Health, Department of Psychiatry, Massachusetts General Hospital and Harvard Medical School, Boston; 2 Neuroendocrine Unit, Department of Medicine, Massachusetts General Hospital and Harvard Medical School, Boston; 3 Division of Women’s Health, Department of Medicine, Brigham and Women’s Hospital, Boston, Massachusetts; 4 Department of Psychiatry, Brigham and Women’s Hospital and Harvard Medical School, Boston, Massachusetts; 5 Eating Disorders Clinical and Research Program, Department of Psychiatry, Massachusetts General Hospital and Harvard Medical School, Boston; 6 Department of Psychiatry, Massachusetts General Hospital and Harvard Medical School, Boston; 7 Department of Pediatrics, Division of Pediatric Endocrinology, Massachusetts General Hospital and Harvard Medical School, Boston

## Abstract

**Importance:**

Individuals with anorexia nervosa maintain extremely low body weights despite elevations in the circulating orexigenic hormone ghrelin. Whether circulating levels of endogenous ghrelin are associated with weight gain in anorexia nervosa is unknown.

**Objective:**

To examine the association between baseline ghrelin and future weight change in individuals with anorexia nervosa.

**Design, Setting, and Participants:**

This prospective cohort study was conducted between April 1, 2014, and March 31, 2020, in the US. Girls and women aged 10 to 22 years were recruited from the greater Boston area from community and area treatment centers, enrolled, and followed up for 18 months. Statistical analyses were performed between January and August 2022.

**Exposures:**

Presence or absence of anorexia nervosa and elevations in endogenous ghrelin.

**Main Outcomes and Measures:**

Changes in age- and sex-standardized body mass index percentiles from baseline to 9- and 18-month follow-up were the main outcomes of interest.

**Results:**

A total of 68 girls and young women (11 [16%] Asian, 4 [6%] Hispanic or Latina, 51 [75%] White [non–Hispanic or Latina], and 2 [3%] other race or ethnicity), including 35 with anorexia nervosa and 33 healthy controls of similar Tanner stage, were included in this study. Anorexia nervosa and healthy control groups were not statistically different by race and ethnicity, Tanner stage, number completing follow-up visits, and the duration between baseline and follow-up visits. At baseline, individuals with anorexia nervosa were slightly older (median [IQR], 20.1 [18.5-21.0] vs 18.7 [14.7-19.4] years; *P* = .005), had lower body mass index percentiles (median [IQR], 2.4 [0.3-4.7] vs 52.9 [40.4-68.3]; *P* < .001), and had elevated circulating ghrelin area under the curve composite index (median [IQR], 1389.4 [1082.5-1646.4] vs 958.5 [743.0-1234.5] pg/mL; *P* = .003) compared with healthy individuals. In linear mixed-effects regression analyses, baseline ghrelin was associated with prospective weight gain after adjusting for diagnosis, age, race, and duration of follow-up (odds ratio, 2.35; 95% CI, 1.43-3.73; *P* = .004).

**Conclusions and Relevance:**

In this cohort study, endogenous ghrelin was associated with longitudinal weight gain in individuals with anorexia nervosa. Further studies are warranted to confirm this result and examine its potential clinical utility in treatment development.

## Key Points

**Question:**

Is there an association between circulating levels of the orexigenic hormone ghrelin and body weight trajectories in individuals with anorexia nervosa?

**Findings:**

In this 18-month cohort study of 68 girls and young women, elevated baseline ghrelin levels were associated with prospective weight gain in anorexia nervosa.

**Meaning:**

This study offers evidence of association between ghrelin and longitudinal weight outcomes in individuals with anorexia nervosa; further studies are warranted to confirm this association and evaluate the potential clinical utility of ghrelin in anorexia nervosa.

## Teaser

This cohort study examines the use of endogenous ghrelin as a baseline factor associated with longitudinal weight trajectories in individuals with anorexia nervosa compared with healthy controls.

## Introduction

Anorexia nervosa (AN) is a debilitating chronic illness with a premature mortality rate 6 times that of the general population—one of the highest among psychiatric illnesses.^1,2^ The mainstay of treatment includes interventions aimed at restoring body weight, which are challenged by illness-driven motivations to remain thin. No medications are currently approved by the US Food and Drug Administration for AN; innovative therapies modulating endocrine signals involved in energy homeostasis, such as ghrelin,^3^ are being investigated.

Ghrelin is a peptide hormone secreted primarily from the stomach that acts on the growth hormone secretagogue receptor 1a in the hypothalamus to promote food intake and weight gain in animal models.^4,5,6^ Surprisingly, even though intravenous ghrelin administration increases food intake in the short term,^7^ elevations in endogenous ghrelin have not been associated with prospective weight gain in humans,^8,9,10^ except in infants born small for gestational age, whose rates of weight gain in the first year of life were positively associated with persistent elevations in ghrelin following glucose administration.^11^ In AN, intravenous ghrelin administration increases food intake,^12^ and endogenous ghrelin levels are significantly elevated,^13^ related to increased secretory bursts.^14^ Whether such high levels of circulating ghrelin have a role in long-term weight homeostasis is unknown. Understanding this association is critical because there is an urgent need for novel therapeutics to assist with weight restoration in this population. In this study, we tested endogenous ghrelin as a baseline factor associated with longitudinal weight trajectory in individuals with AN compared with healthy controls (HCs).

## Methods

This cohort study used data collected as part of a larger parent study examining multidimensional aspects of food motivation pathways, including hormones, neural circuitry, and psychological symptoms, as mediators of eating disorder trajectories for 18 months in female adolescents and young adults with low-weight eating disorders, including AN. A summary of the parent study protocol, including eligibility criteria and study procedures, appears in the eMethods in Supplement 1. This study followed the Strengthening the Reporting of Observational Studies in Epidemiology (STROBE) reporting guidelines.^15^

### Data Collection

Data collection took place between April 1, 2014, and March 31, 2020, at Massachusetts General Hospital (MGH) with institutional review board approval. All participants provided written informed consent or parental consent with assent of minors younger than 18 years. Participants were recruited from clinical programs at MGH and beyond, including the MGH Eating Disorders Clinical and Research Program, specialized treatment centers in the greater Boston area, student health centers at local universities, and community advertisements.

Female participants aged 10 to 22 years were eligible if they met the *Diagnostic and Statistical Manual of Mental Disorders* (Fifth Edition) (*DSM-5*)^16^ criteria for AN with insufficient energy intake relative to requirements due to restrictive eating behaviors characteristic of AN, resulting in a significantly low body weight, or were HCs without a lifetime history of eating disorders with similar Tanner stages. *DSM-5* defines low body weight for adults with AN as body mass index (BMI) less than 18.5 (calculated as weight in kilograms divided by height in meters squared) but does not define a cutoff BMI percentile for children and adolescents with AN, and low body weight in this study was defined as no greater than the 10th BMI percentile for age and sex.^17,18^ Screening for AN included evaluation to confirm the lack of any organic conditions that could account for the low body weight. Study participants had no history of diabetes, gastrointestinal conditions or procedures, recent systemic hormone use, or pregnancy, which could impact ghrelin levels. Participants were medically and psychiatrically stable with sufficiently mitigated imminent risk of harm, without dangerous and active medical issues (eg, severe or rapidly progressing electrolyte imbalances or anemia) or acutely elevated psychiatric risk (eg, active suicidality). See the eMethods in Supplement 1 for details of selection criteria.

Data were collected at baseline, 9-month, and 18-month study visits. Each visit started with anthropometric measurements and a fasting blood draw, after which participants were asked to eat an approximately 400-kcal mixed meal standardized for macronutrient content, with approximately 20% fat, 60% carbohydrates, and 20% protein. At 0.5, 1.0, and 2.0 hours after meal initiation, blood samples were collected. Blood was fractionated to obtain plasma, and plasma total ghrelin was measured using an enzyme-linked immunosorbent assay (interassay and intra-assay coefficients of variation, 6.6%, 1.3%; EZGRT-89K, Millipore Sigma).

### Statistical Analysis

To test the association between baseline ghrelin and longitudinal changes in body weight, we filtered the data set for the availability of (1) body weight measurements at baseline and one or both follow-up visits and (2) baseline ghrelin measurements. We tested our a priori hypothesis with a 2-sided significance level of *P* < .05 using linear mixed models (LMMs) estimating the weight change from baseline to either follow-up visit as a repeated-measures outcome of interest with a random intercept for each individual with ghrelin as the main independent variable of interest.

To compare body weight changes in a developmentally diverse cohort, we derived standardized BMI percentiles and *z* scores using the lambda-mu-sigma (LMS) method to account for the changing skewness of population growth distributions during development, with the participant’s age, sex, measured weight in kilograms, and measured height in meters, together with the US population data.^19^ For individuals older than 20 years (26 of 68 [38%] at baseline, 30 of 63 [48%] at 9 months, and 40 of 61 [66%] at 18 months), population data were extrapolated to derive LMS-standardized BMI percentiles.

The primary outcome of interest, the weight change index, was set a priori as the fold change in BMI percentile from baseline to follow-up (ie, ratio) to account for the significance of weight gain in the at-risk AN population with extremely low BMI percentiles, together with clinical considerations taking into account the challenges in gaining weight for participants with AN vs HCs. As an example, BMI percentile changes from 1 to 5 in participants with AN and from 51 to 55 in HCs both result in an increase of 4, but the fold-change method factors in the clinical significance of weight gain in individuals with low body weights (ie, [5 / 1] × 100 or 500% in AN) vs those with healthy weight (ie, [55 / 51] × 100 or 108% in HCs), respectively. The BMI percentiles (which are always positive) rather than equivalent *z* scores (may be positive, zero, or negative) were used after LMS standardization to compute fold changes using nonzero and nonnegative values.

Dynamic ghrelin measurements across fasting and postprandial time points of the baseline visit were integrated to derive a single composite index (ie, total area under the curve [AUC]) using the trapezoid rule.^20^ Ghrelin AUC was used as the main independent variable of interest for the univariate LMMs. Age, self-reported race, and diagnostic group at baseline visit and duration of follow-up specific to the visit for the weight change index were used as covariates in the primary multivariable LMM. We included race as a variable in this study (and, later in the sensitivity analysis, ethnicity as well) to account for racial (and ethnic) differences in body weight regulation. Included racial groups were American Indian or Alaska Native, Asian, Black or African American, White, and other. Ethnicity groups included HIspanic or Latina and not Hispanic or Latina. Diagnostic group was included as an interactor given clinical considerations of how chronic starvation in AN may result in associated elevations in ghrelin. With ghrelin AUC as the main independent variable, no covariates had significant effects on the model fit, including the interaction term, in a stepwise backward deletion of variables. Covariate terms were scaled to mitigate the effects of any 1 term having an undue influence on the model. To minimize bias, no outliers were removed in the main LMM, and joint assessments of nonindependent covariates (ie, diagnosis and baseline BMI percentiles) were reserved for sensitivity analyses.

Odds ratios (ORs) are easier to interpret than LMM estimates for clinical outcomes such as changes in body weight, which can be dichotomized to derive clinically informative outcome categories (ie, 10% weight gain); however, dichotomization of a continuous outcome can result in loss of information. We derived OR estimates for longitudinal weight gain positively associated with ghrelin AUC directly from LMMs.^21^

To test the robustness of the primary analysis, we performed a complementary set of sensitivity analyses using LMMs with (1) outlier removal, (2) different sets of covariates with ghrelin AUC, (3) same set of covariates with individual ghrelin measurements, (4) alternatively defined outcome variable for longitudinal change in body weight, (5) alternate assumptions of variance estimators, and (6) alternatively defined random-effects terms. Because ORs were derived for fixed-effect terms only, 3 of the 17 LMMs from sensitivity analyses that tested how different random-effect model terms affect the model fit are not shown in the summary table for clarity of presentation of other sensitivity analyses. Each random-effect term was reduced, and likelihood ratio tests of model reductions were assessed with sequential analysis of variance decomposition of random effects. In LMMs with alternate random effect terms, we observed associations consistent with the main LMM (*P* < .05 for all 3 LMMs). Moreover, multivariable linear regression models (LMs) for 9- and 18-month visits were tested to assess the sensitivity of repeated-measures approach in LMMs. In addition, we tested the sensitivity of our approach to derive the ORs directly from LMMs by dichotomizing the continuous outcome with a range of cutoff values for percentage of weight gain in a logistic regression approach with univariate generalized linear mixed-effects models (GLMMs) with baseline ghrelin AUC as the positively associated independent variable.^21^ Lastly, to explore the possibility of large effects of ghrelin AUC diluting effects of associated diagnostic groups in the LMM, we performed subgroup analyses with subsets of data limited to participants with AN or HCs. We limited subgroup analysis to LMMs with 3 terms, with ghrelin AUC and sets of 2 covariate terms from the main model (not including diagnosis).

All multivariable LMMs and LMs were assessed with stepwise backward deletion of single terms to assess how dropping each term would affect model fit. Outliers beyond 2 SDs of the mean weight change index were assessed but not removed except when indicated (ie, sensitivity analysis with outlier removal and models using subsets of data as in simple LMs for each follow-up and LMMs for subgroup analyses) to mitigate the potential for amplified outlier effects in models with fewer data points as well as to prevent uneven outlier effects in models with data subsets, which could introduce undue bias (ie, outliers of weight outcome only in the AN group or greater proportion of outliers at 9 months than 18 months). Similar to the a priori hypothesis tested with the primary analysis, post hoc hypothesis testing for sensitivity and subgroup analyses used a 2-sided significance level of *P* < .05 together with corrections for multiple comparisons using the Benjamini-Hochberg method to mitigate the risk of chance discoveries (type I errors). Even so, sensitivity and subgroup analyses should be interpreted as exploratory given the post hoc nature.

Statistical analyses were performed between January and August 2022 with R and RStudio, versions 4.0.0 and 0.99.902 (R Foundation for Statistical Computing).^22^ Additional details are reported in the eMethods in Supplement 1.

## Results

A total of 198 individuals consented or assented to participate and 76 (38.4%) did not complete the baseline study visit: 47 failed the screen (see reasons in the eMethods in Supplement 1), 11 were lost to follow-up, and 18 withdrew consent or assent before completing the baseline visit. Of the 122 who completed baseline visit procedures, 9 were lost to follow-up and 8 withdrew consent or assent before completing the 9-month study visit, resulting in data from 105 individuals (86.1%) who were followed up longitudinally for at least 9 months. Of those followed up longitudinally, 70 met the criteria for the AN or HC group. Weight or ghrelin data were not available for 2 individuals with AN, resulting in available data from a total of 68 individuals.

### Baseline Characteristics

A total of 68 girls and young women (11 [16%] Asian, 4 [6%] Hispanic or Latina, 51 [75%] White [non–Hispanic or Latina], and 2 [3%] other race or ethnicity), including 35 with AN (median [IQR] age, 20.1 [18.5-21.0] years) and 33 HCs (median [IQR] age, 18.7 [14.7-19.4] years) of similar Tanner stage, were included in this study. The AN group was significantly older than the HC group by 1.6 years (95% CI, 0.5-2.8 years; *P* = .005). The AN and HC groups were comparable in race and ethnicity, Tanner stages, number completing follow-up, and duration of follow-up. For individuals with AN, we observed lower BMI percentiles at all visits (between-group difference for baseline visit, −51.8; 95% CI, −58.8 to −46.1; *P* < .001) (Table 1) and higher baseline visit ghrelin AUC (between-group difference in medians, 327.9 pg/mL; 95% CI, 119.6-543.9 pg/mL × 2 hours; *P* = .003). The AN group gained weight with median BMI percentile fold changes of 1.9 (IQR, 0.6-22.1) at approximately 9 months and 5.8 (IQR, 2.7-36.1) at approximately 18 months, whereas the HC group had relatively stable weights over time with median BMI percentile fold changes of 1.0 (IQR, 0.9-1.1) at approximately 9 months and 1.1 (IQR, 0.9-1.2) at approximately 18 months. See Table 1 for clinical and demographic characteristics of the study participants.

**Table 1.** Study Participant Characteristics

Characteristic	AN (n = 35)	HC (n = 33)	Difference (95% CI)^a^	*P* value^b^
Age, median (IQR), y	20.1 (18.5 to 21.0)	18.7 (14.7 to 19.4)	1.6 (0.5 to 2.8)	.005
Tanner stage, median (IQR)				
Breast development	5 (5 to 5)	5 (4 to 5)	0 (0 to 0)	.17
Genital development	5 (4 to 5)	5 (4 to 5)	0 (0 to 0)	.81
Race and ethnicity^c^				
Other than White				
Any	7 (20)	6 (18)	NA	.79
American Indian or Alaska Native	0	0	NA	
Asian	6 (17)	5 (15)	NA	
Black or African American	0	0	NA	
Other	1 (3)	1 (3)	NA	
White	28 (80)	27 (82)	NA	
Hispanic or Latina	3 (9)	1 (3)	NA	
Not Hispanic or Latina	25 (71)	26 (79)	NA	
Completed visits^d^				
Baseline	35 (100)	33 (100)	NA	.38
9 mo	31 (89)	32 (97)	NA	
Without 9-mo follow-up	4 (11)	1 (3)	NA	
18 mo	31 (89)	30 (91)	NA	
Without 18-mo follow-up	4 (11)	3 (9)	NA	
Both follow-ups	27 (77)	29 (88)	NA	
Duration of follow-up from baseline, median (IQR), mo^d^				
9 mo	9.1 (8.6 to 9.5)	9.1 (8.8 to 9.4)	0.1 (−0.3 to 0.4)	.65
18 mo	18.5 (17.8 to 19.4)	18.0 (17.7 to 18.7)	0.5 (−0.1 to 1.2)	.14
BMI percentile^d^				
Baseline	2.4 (0.3 to 4.7)	52.9 (40.4 to 68.3)	−51.8 (−58.8 to −46.1)	<.001
9 mo	4.7 (1.8 to 13.9)	52.0 (40.4 to 71.1)	−45.0 (−53.1 to −36.7)	<.001
18 mo	11.3 (3.5 to 26.5)	54.0 (43.4 to 69.7)	−40.7 (−49.1 to −30.3)	<.001
Weight change index, fold change in BMI percentile from baseline visit				
9 mo	1.9 (0.6 to 22.1)	1.0 (0.9 to 1.1)	0.9 (0.0 to 4.4)	.06
18 mo	5.8 (2.7 to 36.1)	1.1 (0.9 to 1.2)	4.6 (2.5 to 13.1)	<.001
Baseline visit ghrelin measurements, pg/mL				
Fasting^e^	821.5 (668.4 to 1013.4)	583.5 (374.3 to 852.6)	216.6 (75.7 to 352.9)	.002
Postprandial^e^				
0.5 h	667.0 (563.3 to 814.2) (n = 33)	467.4 (373.9 to 641.6) (n = 32)	167.0 (40.7 to 274.2)	.008
1 h	611.5 (505.1 to 705.8) (n = 30)	475.8 (349.2 to 607.4) (n = 31)	119.9 (19.8 to 224.3)	.02
2 h	608.9 (464.5 to 727.1) (n = 35)	413.4 (325.1 to 604.0) (n = 33)	164.3 (65.4 to 263.9)	.003
Composite AUC, pg/mL × 2 h^f^	1389.4 (1082.5 to 1646.4)	958.5 (743.0 to 1234.5)	327.9 (119.6 to 543.9)	.003

Abbreviations: AN, anorexia nervosa; AUC, area under the curve; BMI, body mass index (calculated as weight in kilograms divided by height in meters squared); HC, healthy control; NA, not applicable.

^a^ Groups were compared using the Wilcoxon rank sum test. Group differences in medians computed with the Hodges-Lehmann estimation are shown with nonparametric 95% CIs, computed as the median of the set of differences between each value in the AN subgroup and each value in the HC subgroup. Two-tailed *P* < .05 was considered significant.

^b^ To compare race and ethnicity, nonzero and nonoverlapping categories of Asian, White Hispanic or Latina, White Not Hispanic or Latina, and other were used with the χ^2^ test. Similarly, to compare number of follow-up completers per visit, nonoverlapping categories of individuals who completed follow-up at month 9 but not at month 18, completed follow-up at month 18 but not at month 9, and completed both follow-up visits were used with the χ^2^ test.

^c^ Race and ethnicity data were self-reported using fixed categories. For non-White individuals, ethnicity breakdown is not shown because all individuals identified as non–Hispanic or Latina.

^d^ Numbers of participants with visit interval and BMI percentile data at respective study visits correspond to the numbers of participants completing each visit.

^e^ Total ghrelin measures at time points surrounding the test meal at baseline visit. Fasting and 2-hour postprandial ghrelin measurements were available for all participants, and sample sizes of available data for 0.5 and 1 hour after the test meal are indicated.

^f^ Composite AUC index for all available ghrelin measurements spanning 2 hours were calculated using the trapezoid method.

### Associations Between Baseline Ghrelin and Longitudinal Weight Trajectory

**Figure 1.**
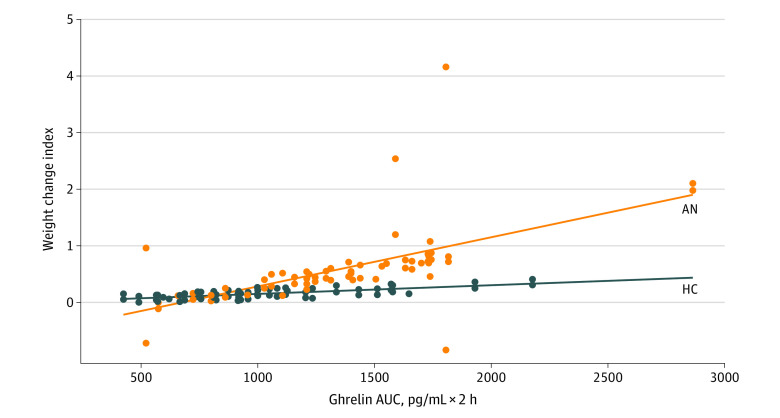
Association Between Baseline Ghrelin and Prospective Change in Body Weight Ghrelin area under the curve (AUC) is plotted against the weight change index, defined as body mass index percentile (surrogate of body weight) at follow-up relative to baseline. Weight change index values shown are model adjusted for age, race, and interval duration between baseline and follow-up. Data from 68 individuals (124 data points) are shown. AN indicates anorexia nervosa; HC, healthy control.

We applied LMMs to estimate the longitudinal weight gain associated with ghrelin AUC. Univariate LMM indicated a statistically significant association (OR, 1.81; 95% CI, 1.29-2.49; *P* = .005). Adjusting for covariation with diagnosis, age, race, and follow-up duration in the main multivariable LMM, the OR of the association was 2.35 (95% CI, 1.43-3.73; *P* = .004). We observed no statistically significant associations for the covariate terms in the main LMM. Results of the main LMM are in Figure 1; raw data not adjusted for covariation appear in eFigure 1 in Supplement 1.

We tested the validity and robustness of the multivariable LMM assessing our a priori hypothesis through an exploratory series of complementary sensitivity analyses. We found statistically significant positive associations between baseline ghrelin and longitudinal weight gain in all 27 regression models (Table 2). Validity of deriving ORs from LMMs was assessed with GLMMs with dichotomization of the outcome with 21 different weight cutoffs (eFigure 2 in Supplement 1).

**Table 2.** Sensitivity Analyses for the Final Model^a^

Regression model	OR (95% CI)	*P* value
Reference		
Main model		
Univariate	1.81 (1.29-2.49)	.005
Multivariable^b^	2.35 (1.43-3.73)	.004
Outlier removal in the data set		
Outliers − SD method^c^	4.49 (2.61-7.34)	<.001
Ghrelin measurements instead of ghrelin AUC		
Fasting	2.17 (1.31-3.50)	.01
0.5 h	2.81 (1.70-4.48)	<.001
1 h	4.27 (2.47-7.00)	<.001
2 h	2.17 (1.32-3.47)	.01
Alternatively defined weight change indexes as outcome of interest		
BMI		
*z* Score delta change	2.63 (1.60-4.20)	<.001
Percentile delta fold change	2.35 (1.43-3.73)	.004
Percentile log fold change	2.96 (1.79-4.74)	<.001
Alternative sets of covariates		
Tanner stage		
By breast development instead of age	2.43 (1.49-3.85)	.002
By genital development instead of age	2.42 (1.49-3.82)	.002
Ethnicity		
Instead of race	2.50 (1.53-3.97)	.002
With race as nonoverlapping categories	2.37 (1.45-3.77)	.004
BMI percentiles at baseline instead of diagnostic group	2.31 (1.44-3.61)	.003
Alternative assumptions of variance estimators		
Restricted maximum likelihood instead of maximum likelihood	2.35 (1.43-3.73)	.006
Linear models using data from specified follow-up visit only^d^		
Ghrelin at 9 mo		
Fasting	5.28 (2.31-10.67)	<.001
0.5 h	10.77 (4.35-22.21)	<.001
1 h	8.42 (3.43-17.37)	<.001
2 h	11.52 (4.65-23.80)	<.001
AUC	8.32 (3.51-16.84)	<.001
Ghrelin at 18 mo		
Fasting	2.42 (1.14-4.81)	.02
0.5 h	3.29 (1.55-6.36)	.001
1 h	2.60 (1.22-5.11)	.01
2 h	3.83 (1.74-7.62)	<.001
AUC	3.02 (1.43-5.90)	.003

Abbreviations: AUC, area under the curve; BMI, body mass index (calculated as weight in kilograms divided by height in meters squared); OR, odds ratio.

^a^ *P* values are from the Wald χ^2^ test for the fixed effects of respective ghrelin term in the linear mixed-effects regression model (LMM) and are not adjusted for multiple comparisons. Multiple comparisons adjustment of the false discovery rate did not change results. For all LMMs in sensitivity analyses, except when testing for effects of removing the outliers, no outliers were removed to avoid introducing bias.

^b^ All parameters were kept the same as the reference main multivariable model of the study with single change as described for each sensitivity analysis.

^c^ From the data set with 124 data points, 6 outlier weight change indexes (<5%) from individuals with anorexia nervosa (AN) were removed before modeling. For 1 individual, data from both visits were identified as outliers; for 3 individuals, data from only 1 follow-up visit were identified as outliers; and for 1 individual who only had single follow-up data, this was identified as an outlier, resulting in the final sample size of 33 for the AN subgroup. No healthy control participants had weight change values in the extremes (n = 33).

^d^ Linear regression models (LMs) were used to confirm the findings from the LMMs using weight change index at a single follow-up visit only, adjusted for diagnostic group, age, race, and follow-up duration. The LMs were built with a data set after removal of the 6 outlier data points before performing the analyses. There were 4 and 2 outlier data points in the 9- and 18-month follow-up data with the final data set from 59 and 61 individuals, respectively.

**Figure 2.**
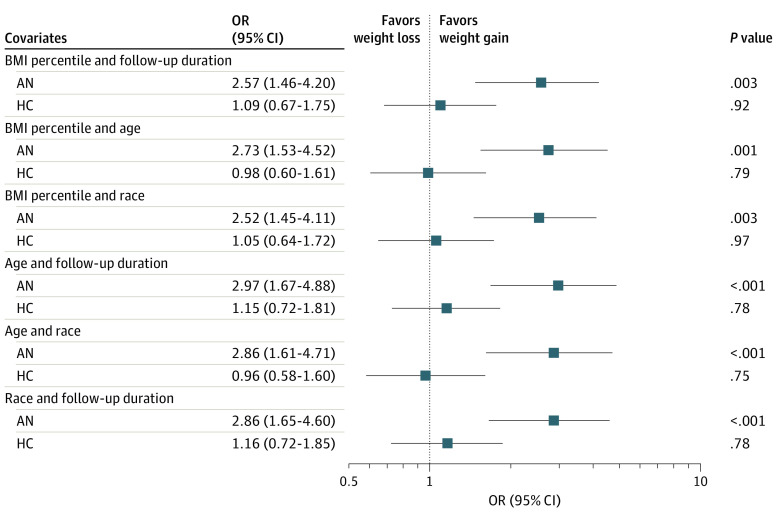
Subgroup Analyses of Baseline Ghrelin and Prospective Change in Body Weight In models stratified by subgroups, ghrelin area under the curve (AUC) was positively associated with future weight gain only in the anorexia nervosa (AN) subgroup, unadjusted and after adjusting for any 2 combinations of the covariates from the final model. Outliers were removed for subgroup analyses resulting in data points from 33 participants with AN and 33 healthy controls (HCs). Subgroup models could not be adjusted for the same set of covariates in the main 5-term model given the sample sizes. Given the exploratory nature of subgroup analysis, analysis expanded the hypothesis testing to include linear mixed models (LMMs) that replace ghrelin AUC with individual ghrelin measurements, which are shown in eFigure 3 in Supplement 1. Findings from the 60 LMMs were consistent with the models with ghrelin AUC term as shown here. BMI indicates body mass index.

Lastly, we conducted subgroup analyses to test the association between diagnostic group and weight trajectory that may have been overshadowed by ghrelin AUC in the main model. We found statistically significant positive associations between ghrelin and longitudinal weight gain only in the AN subgroup and not for HCs, which was a consistent finding across 60 LMMs tested for subgroups (12 LMMs with ghrelin AUC in Figure 2; 48 other LMMs in eFigure 3 in Supplement 1), with no changes after correction for multiple comparisons (false discovery rate *P* < .025).

## Discussion

This prospective cohort study found that baseline ghrelin was positively associated with longitudinal weight gain in girls and young women with AN in community settings during approximately 18 months of follow-up. Of note, robust associations in our extensive series of sensitivity analyses support the validity of results regardless of modeling with ghrelin measurements from fasting or postprandial time points or the composite index and regardless of how longitudinal change in body weight is defined. Although the primary analysis did not find statistically significant results of the diagnostic grouping jointly with ghrelin, exploratory subgroup analyses support the specificity of association only in AN.

Our finding that ghrelin is associated with longitudinal weight gain in AN is in stark contrast to prior longitudinal investigations in other human populations, and this is the first, to our knowledge, to report this association in those older than infants. Prior studies^8,9,10^ have consistently demonstrated the lack of association between ghrelin and weight trajectory despite the short-term effects of ghrelin in stimulating food intake in humans^7^ and preclinical evidence for a weight regulatory role of ghrelin. The only prior human study^11^ that observed a positive association between ghrelin and prospective weight gain was in infants born small for gestational age, with the association observed only with ghrelin measurements following glucose administration but not for fasting ghrelin. Although we observed robust associations across fasting and postprandial time points, we note that AN and small for gestational age cohorts have low body weights relative to expected for developmental stage based on population norms. It is possible that having very high levels of circulating ghrelin in AN (an observation in agreement with prior studies^13^) together with vulnerability from extremes of low body weights may exert an effect on energy homeostasis and modulate weight-regulatory mechanisms in this vulnerable population.

Large-scale genomic investigations have identified heritable risk factors for AN, which implicate genetic loci associated with metabolic regulation.^23^ Ghrelin is known to have multifaceted roles in regulating glucose metabolism and is being investigated as a potential new avenue of therapeutics development for metabolic diseases such as type 2 diabetes.^24^ In addition, genes encoding components of the ghrelin signaling pathway have been studied as risk factors for AN, with 1 study^25^ reporting that specific single-nucleotide polymorphisms of the ghrelin gene were associated with longitudinal weight recovery in AN. As such, effects of ghrelin signaling may be associated with the course of illness in AN, but this should be interpreted with caution because evidence regarding effects of ghrelin signaling on longitudinal weight gain remains limited.

For instance, a prior randomized clinical trial^3^ evaluated the effects of a ghrelin receptor agonist, relamorelin, on body weight outcome for 22 women with AN (mean age, 28.9 years). Ghrelin agonist treatment resulted in greater weight increases after 4 weeks compared with placebo, with means (SEMs) of 0.86 (0.40) and 0.04 (0.28) kg, but between-group difference in means was not statistically significant (*P* = .07). This finding is in line with our findings regarding the positive association between endogenous levels of ghrelin and longitudinal weight gain. Even so, it remains unknown whether ghrelin signaling has a direct effect on weight gain in AN, and additional studies to understand ghrelin biology specific to AN are warranted given the urgent need for new treatments because of the lack of US Food and Drug Administration–approved medications for this illness, which has one of the highest mortality rates in psychiatry.

### Limitations

This study has some limitations. Although the cohort age range spans the full developmental spectrum when AN typically presents, the generalizability of our results is limited by the relatively modest sample size and lack of racial, ethnic, and gender diversity. Larger longitudinal studies are needed to confirm the association of elevated endogenous ghrelin with weight trajectory in AN. We also acknowledge the limitations in deriving ORs from LMMs, because the method was developed without accounting for the variance of random effects, which could result in an overestimation in applications to LMMs despite the benefits over GLMMs. This limitation is mitigated by rigorous hypothesis testing directly with LMMs together with extensive and complementary sensitivity analyses, including a series with GLMMs that support the validity and robustness of our findings. Nevertheless, the use of ORs was intended to enhance the interpretability of the results for an outcome variable critical to AN in clinical settings.

## Conclusions

In this cohort study, circulating levels of ghrelin in adolescent girls and young women with AN were positively associated with prospective weight gain during 18 months of follow-up. This study provides evidence supporting further research into the role of ghrelin biology in weight regulation in AN.

## Notes

Supplement 1. **eMethods.** Supplemental Methods **eFigure 1. **Association Between Baseline Ghrelin and Prospective Change in Body Weight Without Adjustment for Covariates **eFigure 2. **Sensitivity Analysis for Deriving Odds Ratios From Linear Regression Models **eFigure 3. **Expanded Subgroup Analyses of Baseline Ghrelin and Prospective Change in Body Weight

Supplement 2.
Data Sharing Statement
