# Supplementary material for: Association Between Ghrelin and Body Weight Trajectory in Individuals With Anorexia Nervosa
Source: JAMA Netw Open. 2023 Mar 24;6(3):e234625. doi: 10.1001/jamanetworkopen.2023.4625 (PMC10313149; doi:10.1001/jamanetworkopen.2023.4625)
Supplement: Supplement 1. — eMethods. Supplemental Methods eFigure 1. Association Between Baseline Ghrelin and Prospective Change in Body Weight Without Adjustment for Covariates eFigure 2. Sensitivity Analysis for Deriving Odds Ratios From Linear Regression Models eFigure 3. Expanded Subgroup Analyses of Baseline Ghrelin and Prospective Change in Body Weight [file jamanetwopen-e234625-s001.pdf]

Supplementary Online Content

Kim YR, Lauze MS, Slattery M, et al. Association between ghrelin and body weight trajectory in individuals with anorexia nervosa. *JAMA Netw Open*. 2023;6(3):e234625. doi:10.1001/jamanetworkopen.2023.4625

eMethods. Supplemental Methods

eFigure 1. Association Between Baseline Ghrelin and Prospective Change in Body Weight Without Adjustment for Covariates

eFigure 2. Sensitivity Analysis for Deriving Odds Ratios from Linear Regression Models

eFigure 3. Expanded Subgroup Analyses of Baseline Ghrelin and Prospective Change in Body Weight

This supplementary material has been provided by the authors to give the readers additional information about their work.

© 2023 Kim YR et al. *JAMA Network Open.*

eMethods

1. Parent Study Protocol

1.1. Parent Study Overview

Findings in this manuscript are derived from data obtained in a larger prospective cohort study aimed at examining multidimensional aspects of abnormal homeostatic and hedonic food motivation pathways including hormones, neural circuits, and psychological symptoms as predictors of eating disorder trajectories over 18 months among girls and young women with low-weight eating disorders, including anorexia nervosa (AN), other specified feeding and eating disorders–atypical AN (OSFED), avoidant restrictive food intake disorder (ARFID), compared to healthy controls with no lifetime history of eating disorders (HC).

1.2. Parent Study Hypotheses

Hypothesis 1: Altered food motivation pathways (ie, hormones, neural circuits, and psychological symptoms) underly patterns of restriction, binge eating, and purging, and differentiates adolescents with low-weight eating disorders from healthy, normal-weight controls.

Hypothesis 2*: Within adolescents with low weight eating disorders followed longitudinally, 18-month outcome (ie, dietary restriction, binge/purge frequency, and weight changes) is determined by food motivation pathways (ie, hormones, neural circuits, and psychological symptoms) at baseline and changes over time.

* Drs. Eddy, Misra, Lawson, and Kim decided to focus on **ghrelin at baseline** as independent variable of interest and **weight changes in AN vs. HC** as the primary outcome of interest, *a priori* to data analysis, to allow for a focused analysis avoiding overlaps with ongoing projects in our team.

1.3. Participant Selection Criteria

Females with low-weight eating disorders (LWED)

Inclusion criteria

1. Female age 10 to 22 years old
2. LWED characterized by (i) low body weight defined as less than 90% of median BMI for age and sex, or less than 90% of ideal body weight for height (IBW, defined as the weight corresponding to the participant's height percentile for age/bone age), with (ii) restrictive eating, binge eating more than once a month, purging more than once a month, excessive exercise more than once a month, and/or in treatment for an eating disorder, to be determined as part of the initial clinical assessment.

Exclusion criteria

1. Use of systemic hormones, pregnancy or breastfeeding within eight weeks
2. Use of Depo-Provera within three months
3. History of psychosis by Schedule for Affective Disorders and Schizophrenia for School Age Children-Present and Lifetime Version (KSADS-PL)
4. Substance or alcohol use disorder active within the past month by KSADS-PL
5. Diabetes mellitus
6. Hematocrit (Hct) < 30.0%
7. Potassium (K) < 3.0 mmol/L
8. Gastrointestinal tract surgery (including gastrectomy, gastric bypass surgery, and small or large bowel resection)
9. Other medical explanation for low weight (e.g., brain tumor)
10. Active suicidal ideation

Healthy control females

Inclusion criteria

1. Female age 10 to 22 years old
2. BMI percentile for age and sex between 25th and 85th percentile
3. Matched to eating disorder cohort to be within 1 Tanner stage
4. Having regular menses for those more than 2 years from menarche
5. No pubertal delay (pubertal delay includes menarche at > 16 years, thelarche at > 13 years).

Exclusion criteria

1. Use of systemic hormones, pregnancy or breastfeeding within eight weeks
2. Use of Depo-Provera within three months
3. History of lifetime psychiatric diagnoses by KSADS-PL
4. Diabetes mellitus
5. Hematocrit (Hct) < 30.0%
6. Potassium (K) < 3.0 mmol/L
7. Gastrointestinal tract surgery (including gastrectomy, gastric bypass surgery, and small or large bowel resection)
8. Active suicidal ideation
9. Excessive exercise within the last 3 months (having run > 25 miles in any one week or having exercised >10 hours in any one week)

© 2023 Kim YR et al. *JAMA Network Open.*

10. Vegetarianism
11. Familial history of Anorexia Nervosa or other low weight eating disorders in first degree relatives

Of note, study definition for LWED was designed to identify individuals with AN meeting the Diagnostic and Statistical Manual of Mental Disorders 5th Edition (DSM-5) criteria, with a cutoff of <90% IBW. As the DSM-5 does not define a cutoff BMI percentiles for children and adolescents with AN. Clinically meaningful cutoffs for BMI percentiles are discussed in Andersen SB, et al. Grasping the weight cut-off for anorexia nervosa in children and adolescents. *Int J Eat Disord*. 2018;51: 1346-1351. In addition to data shown in our manuscript, our study enrolled a *subset* of participants meeting DSM-5 criteria for other specified feeding and eating disorders, atypical AN (OSFED) and ARFID, and having low body weight by the BMI percentile cutoff.

1.4. Study Procedures

Individuals presented to morning visits in their fasting states.

Pre-meal procedures overview

1. History and physical exam with evaluation of height, weight, Tanner stage.
2. Assessments of dietary habits and physical activity levels with 4-day food record and Paffenbarger Questionnaire.
3. Blood draw for fasting hormones, including ghrelin, peptide YY (PYY), cholecystokinin (CCK), oxytocin, brain-derived neurotrophic factor (BDNF), estradiol, progesterone, triiodothyronine (T3), T3 uptake, total thyroxine (T4), free T4, thyroid stimulating hormone (TSH), leptin, cortisol, insulin-like growth factor 1 (IGF-1).
4. MRI pre-meal with presentation of food and non-food visual stimuli
5. Psychological symptom reports pre-meal
    a. Appetite Visual Analog Scales (VAS) 1 hour pre-meal and within 5 minutes pre-meal.
    b. One time pre-meal Power of Food Scale and Temporal Experience of Pleasure Scale

Study meal

Subjects were asked to eat an approximately 400-kilocalorie mixed meal standardized for macro-nutrient content (approximately 20% fat, 60% carbohydrates, and 20% protein).

Post-meal procedure overview

1. Serial blood draws for postprandial hormone assessments of ghrelin, PYY, CCK, oxytocin, BDNF.
2. MRI 30 minutes post-meal with presentation of food and non-food visual stimuli
3. Psychological symptom reports post-meal
    a. Appetite VAS within 5 minutes post-meal and 1 hour post-meal.
    b. Quantification of food intake.

Behavioral questionnaires and assessments used in screening and/or study visits

1. Eating Inventory (EI)
2. Eating Expectancy Inventory (EEI)
3. Eating Disorder Inventory-3 (EDI-3)
4. Behavioral Inhibition System/Behavioral Approach System (BIS/BAS)
5. Cookie Taste Test
6. Delayed Discounting Task
7. Eating Disorder Examination (EDE)
8. Eating Disorders Longitudinal Interval Follow-up Evaluation 3 (LIFE-EAT 3)
9. Implicit Association Test (IAT)
10. Child Development Inventory (CDI-2)
11. Beck Depression inventory II (BDI-II)
12. State Trait Anxiety Inventory for Children (STAI-C)/State Trait Anxiety Inventory (STAI)
13. Toronto Alexithymia Scale (TAS-20)
14. Liebowitz Social Anxiety Scale (LSAS-SR)
15. Alcohol Use Disorders Identification Test (AUDIT)
16. Drug Use Questionnaire (DAST-10)

Optional tests with separate consent procedures

1. Saliva and blood collection for DNA
2. Hair collection for hair cortisol
3. Stool collection for microbiome analysis
4. Dual-energy X-ray absorptiometry (DXA) for bone mass density and body composition

© 2023 Kim YR et al. *JAMA Network Open.*

eMethods

2. Reasons for Screen Failures

Reasons for screen failures for individuals screened as low-weight eating disorder (LWED) or healthy controls (HC) included:

- Not meeting BMI criteria for 5 LWED and 3 HC.
- Not meeting the eating habits criteria for 7 LWED and 2 HC.
- Related to psychiatric symptoms, with one individual with LWED who was found to have active substance use disorder and 13 screened as HC found to have active or lifetime history of psychiatric illnesses
- Other reasons excluded 4 LWED and 2 HC.

Individuals screened out may not have gotten a full diagnostic classification with regards to the presence or absence of AN and LWED term is used above.

© 2023 Kim YR et al. *JAMA Network Open.*

eMethods

3. Statistical Analyses

3.1. Statistical Environment

R and RStudio platforms were used for statistical analyses with the following R packages: tidyverse, lme4, lmerTest, car, performance, broom, lubridate, psych, emmeans, ggplot2, visreg, and forestploter. *JAMA* color scheme for figures was obtained using ggsci library.

3.2. Descriptive Data

Data distribution was assessed by the Shapiro-Wilk test and non-parametric data from AN and HC were compared using the Wilcoxon rank sum test. Group differences in medians were computed with the Hodges-Lehmann estimation are shown with non-parametric 95% confidence interval, computed as the median of the set of differences between each value in AN and each value in HC. Contingency tables were analyzed by chi-squared test with Yates continuity correction for goodness of fit. Two-tailed $P<0.05$ was considered significant.

3.3. Primary Analysis

The main hypothesis was tested using linear mixed-effects regression model (LMM) estimating longitudinal weight change index as a factor of baseline visit ghrelin AUC through the *lmer* function from lme4 library in R. Repeated-measures data of fold change in BMI percentiles by either 9 or 18 months as response/outcome of interest was fit using LMM with fixed effects for slopes and intercepts and random effects of the individuals as random intercepts, with the term (1 | id), where id represents study id for the participants. Visit interval and outcome of interest were scaled without centering.

3.4. Secondary Analyses

Sensitivity analyses assessed the robustness of primary analysis testing *a priori* hypothesis.
Multivariate LMMs with each of the below as the single change to the main LMM were tested:

- Removal of outliers beyond 2 standard deviation units of the mean
- Individual ghrelin measurements as alternatives to the main independent variable of interest (ie, ghrelin AUC)
  - Fasting ghrelin
  - Postprandial ghrelin 0.5 h after meal initiation
  - Postprandial ghrelin 1 h after meal initiation
  - Postprandial ghrelin 2 h after meal initiation
- Alternatives to the main outcome variable of interest (ie, weight change index defined as fold change in BMI percentile from baseline to follow-up visit)
  - BMI z-score delta change, calculated as: (BMI z-score at follow-up – BMI percentile at baseline)
  - BMI percentile delta fold change, calculated by dividing the BMI percentile delta by baseline value: ((BMI percentile at follow-up – BMI percentile at baseline) / BMI percentile at baseline)
  - BMI percentile log fold change, calculated by: ($\log_{10}$(BMI percentile at follow-up / BMI percentile at baseline))
- Alternative sets of covariates, replacing the main model's covariate terms with those not included in the main model to account for the non-independence of variables (ie, Tanner staging and age, race and ethnicity, presence or absence of AN diagnosis and BMI percentile)
  - Tanner stages by breast development instead of age, dichotomized at or below stage 5
  - Tanner stages by genital development instead of age, dichotomized at or below stage 5
  - Ethnicity instead of race
  - Ethnicity with race as nonoverlapping categories as the only individuals identifying as Hispanic were white (ie, White Hispanic, White Not Hispanic, Asian Not Hispanic, and Other Not Hispanic)
  - Baseline BMI percentiles instead of diagnostic group
- Alternate model assumptions regarding variance estimators, to account for the possibility that the main LMM solved using maximum likelihood (ML) principle may under or overestimate the true variance, final model was also tested with the restricted or residual maximum likelihood (REML) method.

Alternative sets of random effect terms were assessed with stepwise deletions of single terms, including:

- Intercept varying among individuals and in 9- or 18-month follow-up visits
- Random intercept and slope by different duration of follow-up and the individual
- Random intercept of the individuals and random slope varying by different duration of the follow-up for 9- or 18-month follow-up visits

In addition, weight change outcome at *either* follow-up was modeled individually using simple linear regression models (LM) without repeated-measures outcome as in LMM approach, with adjustment for the same covariate terms of the main model. Given the exploratory nature of LMs, we expanded this to build LMs with each of the ghrelin measurements in addition to the AUC index.

© 2023 Kim YR et al. *JAMA Network Open.*

Sensitivity of deriving odds ratios (ORs) directly from the linear model without dichotomizing the continuous outcome variable was assessed by dichotomizing the weight change index into binary outcomes of presence or absence of weight gain at or above specified percentage, to derive ORs of generalized linear mixed effects logistic regression models (GLMMs).

Lastly, subgroup analysis was performed as described in the main text. Given the exploratory nature of LMMs built for each subgroup, we expanded this approach by building LMMs with each of the ghrelin measurements in addition to the AUC index.

3.5. Handling of Outliers

Outliers of weight change outcome were identified by filtering for values beyond 2 standard deviation units of the mean. From the dataset with 124 datapoints, 6 outlier weight change indexes (<5%) from 5 individuals with anorexia nervosa were identified. For one individual, data from both visits were identified as outliers; for 3 individuals, only one of the follow-up visit data was identified as outliers; and for one individual who only had a single follow-up data, this was identified as an outlier, resulting in the final n=33 in this dataset. No healthy control participants had weight change values in the extremes (n=33 for HC). The dataset without outliers, with 118 datapoints from N=66 individuals, was used in LMs for each follow-up visit, LMMs for subgroup analyses, and LMM for sensitivity testing for outlier removal.

© 2023 Kim YR et al. *JAMA Network Open.*

eFigure 1. Association Between Baseline Ghrelin and Prospective Change in Body Weight Without Adjustment for Covariates.

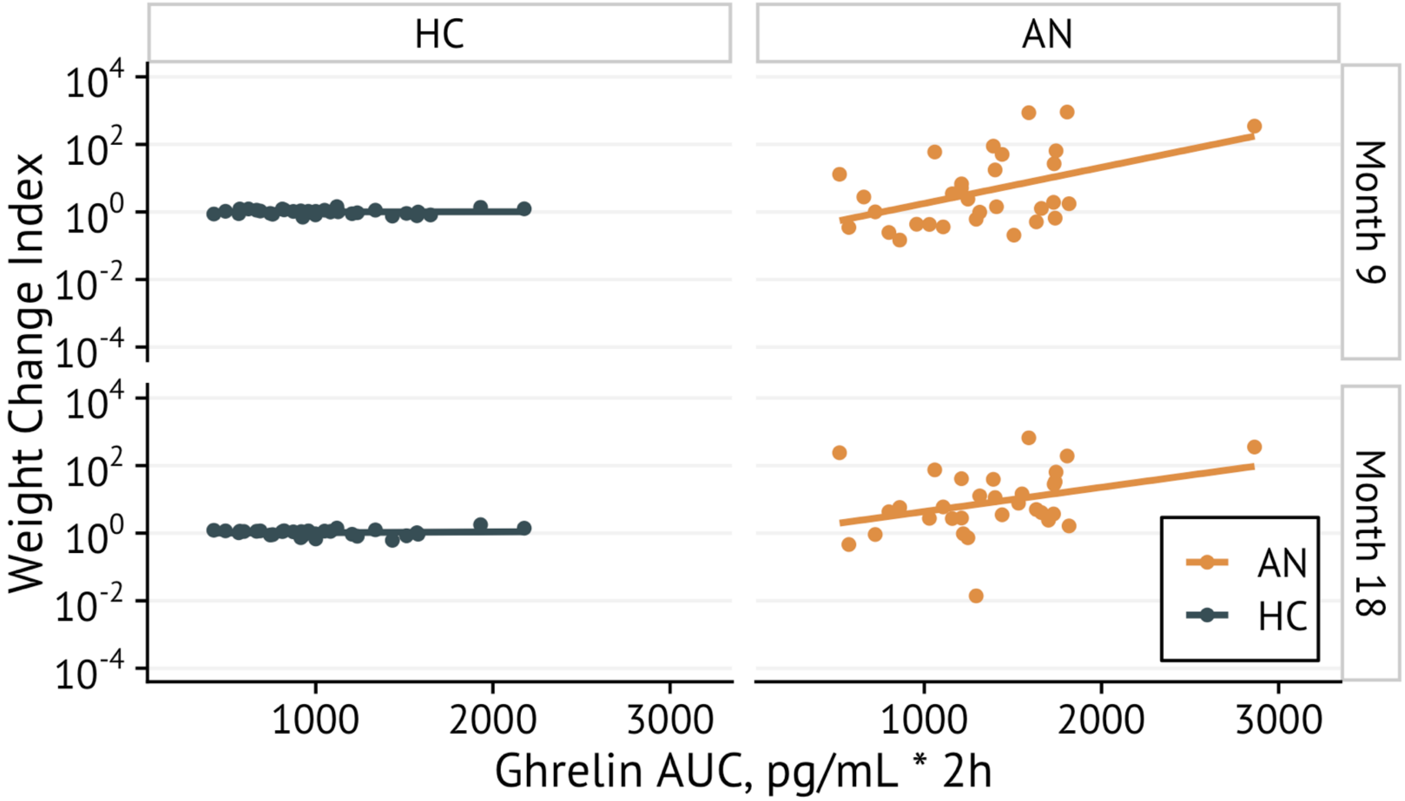

Abbreviations: AN, participants with anorexia nervosa; HC, healthy control participants; AUC, area under the curve.

Ghrelin AUC data from N=68 participants is plotted with unadjusted Weight Change Index values at respective follow-up visit grouped by diagnosis and with y-axis in exponential scale.

© 2023 Kim YR et al. *JAMA Network Open.*

eFigure 2. Sensitivity Analysis for Deriving Odds Ratios from Linear Regression Models

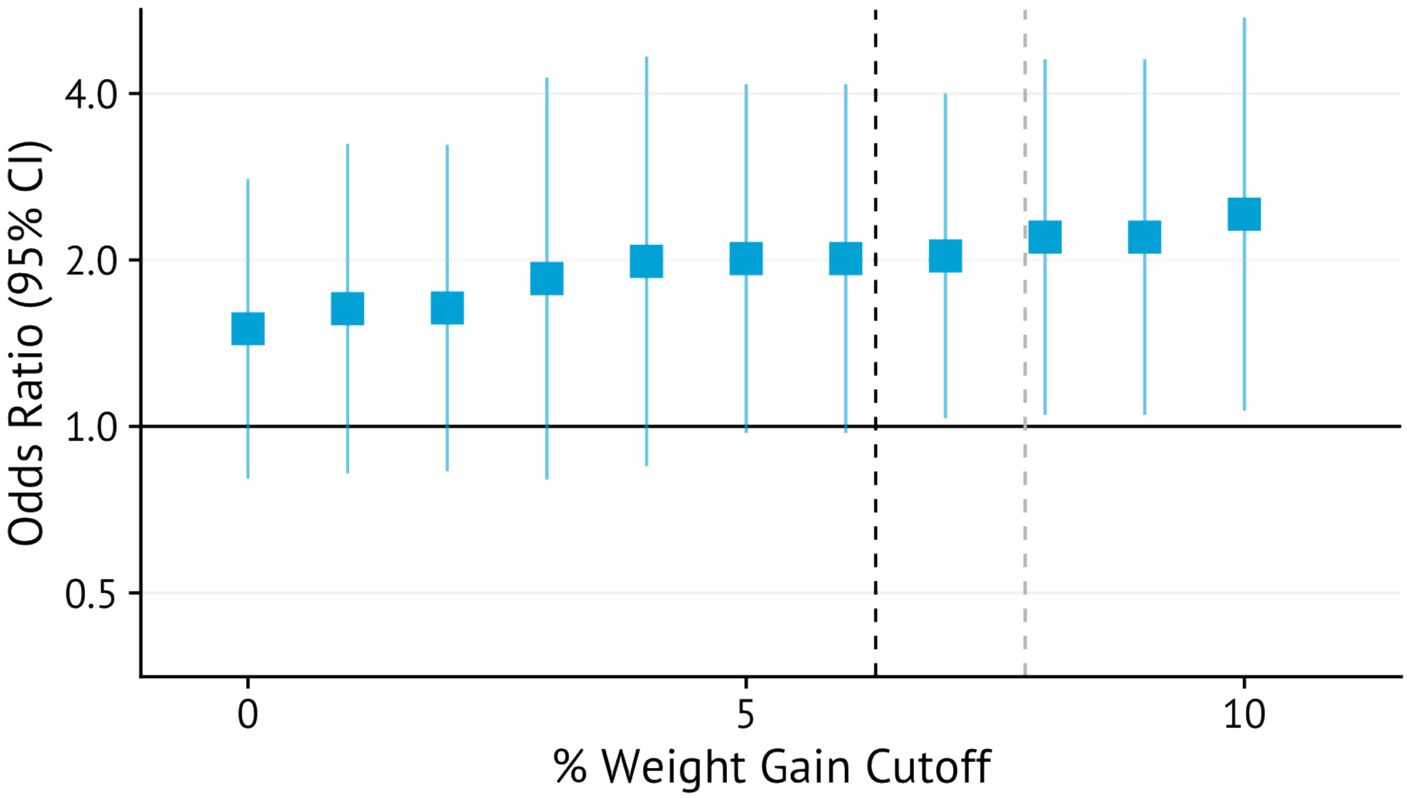

Sensitivity of deriving odds ratios (ORs) directly from linear models *without* dichotomizing the continuous outcome was assessed by exploratory analysis of ORs derived *with* dichotomization of the outcome into binary groups to build univariate logistic regression models, or generalized linear mixed effects models (GLMM). Binary outcome groups were the presence or absence of weight gain at or above the specified cutoff % weight gain, the ORs of which were estimated to test the association between ghrelin AUC and future weight gain. For a total of 21 GLMMs with cutoff points ranging from 0 to 20% increases in body weight, univariate GLMM ORs of gaining 7 or higher % weight (ie, cutoff values 7 through 20) were positively associated with ghrelin AUC. After accounting for multiple comparisons with Benjamini-Hochberg method with a false discovery rate of .038, cutoff values 8 through 20 resulted in statistically significant associations. Figure shows the model results with cutoff % weight gain ranging from 0 to 10% and unadjusted and adjusted significance thresholds are indicated with black and grey dashed lines, respectively.

© 2023 Kim YR et al. *JAMA Network Open.*

eFigure 3. Expanded Subgroup Analyses of Baseline Ghrelin and Prospective Change in Body Weight

A. Ghrelin, Fasting

	AN		HC	
Covariates	OR (95% CI)	P value	OR (95% CI)	P value
BMI percentile and follow-up duration	2.04 (1.19 to 3.32)	0.02	1.01 (0.63 to 1.62)	0.93
BMI percentile and age	2.11 (1.22 to 3.46)	0.01	0.92 (0.57 to 1.49)	0.65
BMI percentile and race	2.00 (1.17 to 3.24)	0.02	0.98 (0.61 to 1.59)	0.83
Age and follow-up duration	2.32 (1.34 to 3.76)	0.005	1.07 (0.68 to 1.69)	0.97
Age and race	2.22 (1.28 to 3.62)	0.009	0.92 (0.57 to 1.50)	0.63
Race and follow-up duration	2.27 (1.34 to 3.62)	0.005	1.08 (0.68 to 1.71)	0.94

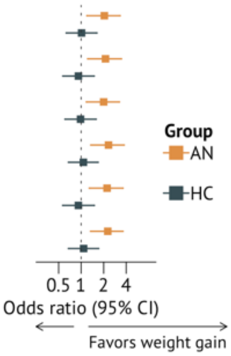

B. Ghrelin, 30 Minutes

	AN		HC	
Covariates	OR (95% CI)	P value	OR (95% CI)	P value
BMI percentile and follow-up duration	2.85 (1.58 to 4.77)	0.001	1.14 (0.70 to 1.83)	0.84
BMI percentile and age	2.96 (1.62 to 4.95)	<0.001	1.06 (0.65 to 1.72)	0.97
BMI percentile and race	2.84 (1.58 to 4.73)	0.001	1.05 (0.64 to 1.73)	0.96
Age and follow-up duration	3.28 (1.81 to 5.42)	<0.001	1.25 (0.78 to 1.97)	0.60
Age and race	3.18 (1.76 to 5.28)	<0.001	1.02 (0.62 to 1.69)	0.88
Race and follow-up duration	3.24 (1.82 to 5.27)	<0.001	1.19 (0.73 to 1.92)	0.74

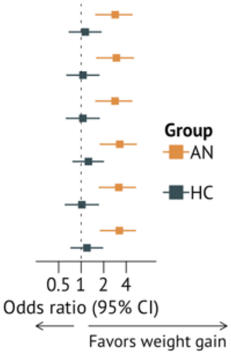

C. Ghrelin, 60 Minutes

	AN		HC	
Covariates	OR (95% CI)	P value	OR (95% CI)	P value
BMI percentile and follow-up duration	2.45 (1.36 to 4.11)	0.007	1.12 (0.70 to 1.80)	0.86
BMI percentile and age	2.85 (1.53 to 4.84)	0.002	1.00 (0.61 to 1.64)	0.83
BMI percentile and race	2.54 (1.40 to 4.26)	0.005	1.05 (0.64 to 1.71)	0.95
Age and follow-up duration	2.90 (1.56 to 4.91)	0.001	1.14 (0.70 to 1.84)	0.84
Age and race	2.93 (1.58 to 4.98)	0.001	0.90 (0.53 to 1.52)	0.59
Race and follow-up duration	2.87 (1.59 to 4.75)	<0.001	1.15 (0.71 to 1.85)	0.81

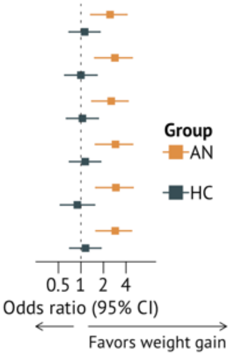

© 2023 Kim YR et al. *JAMA Network Open.*

D. Ghrelin, 120 Minutes

	AN		HC	
Covariates	OR (95% CI)	P value	OR (95% CI)	P value
BMI percentile and follow-up duration	3.00 (1.64 to 5.05)	0.001	1.22 (0.75 to 1.95)	0.61
BMI percentile and age	3.05 (1.66 to 5.14)	<0.001	1.11 (0.68 to 1.81)	0.86
BMI percentile and race	2.80 (1.56 to 4.65)	0.001	1.20 (0.74 to 1.93)	0.67
Age and follow-up duration	3.56 (1.95 to 5.90)	<0.001	1.27 (0.79 to 2.00)	0.50
Age and race	3.30 (1.81 to 5.48)	<0.001	1.13 (0.69 to 1.83)	0.79
Race and follow-up duration	3.34 (1.88 to 5.44)	<0.001	1.30 (0.81 to 2.05)	0.45

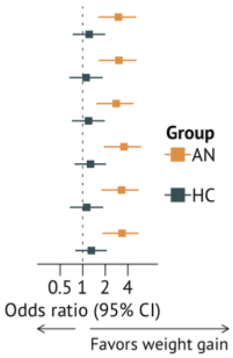

Abbreviations: OR, odds ratio; AN, anorexia nervosa; HC, healthy control.

In models stratified by subgroups, individual ghrelin measurements at different timepoints were positively associated with future weight gain only in AN subgroup, unadjusted and after adjusting for any two combinations of the covariates from the main model. Outliers were removed for subgroup analyses resulting in sample sizes as follows: 33 and 33 at fasting state; 32 and 32 for 0.5 h, 29 and 31 for 1 h, and 33 and 33 for 2 h timepoints, for the AN and HC groups, respectively.

Subgroup models could not be adjusted for the same set of covariates in the main 5-term model given the sample sizes. Odds ratios are summarized as a forest plot with axis in logarithmic scale. 3A through 3D shows the results of LMMs using individual ghrelin measurements rather than ghrelin AUC, findings which appear in **Figure 2**. For a total of 60 multivariate LMMs 3 terms in the subgroup analysis (ie, adjusting ghrelin term with 6 sets of two other covariates from the main model, together with each of 5 different ghrelin terms for AN and for HC), ghrelin was positively associated with longitudinal weight outcome after accounting for multiple comparisons with Benjamini-Hochberg method (FDR < .025).

© 2023 Kim YR et al. *JAMA Network Open.*
