# Supplementary material for: Association Between Ghrelin and Body Weight Trajectory in Individuals With Anorexia Nervosa
Source: JAMA Netw Open. 2023 Mar 24;6(3):e234625. doi: 10.1001/jamanetworkopen.2023.4625 (PMC10313149; doi:10.1001/jamanetworkopen.2023.4625)
Supplement: Supplement 2. — Data Sharing Statement [file jamanetwopen-e234625-s002.pdf]

Data Sharing Statement

Kim. Association Between Ghrelin and Body Weight Trajectory in Individuals With Anorexia Nervosa. *JAMA Netw Open*. Published March 24, 2023.
doi:10.1001/jamanetworkopen.2023.4625

Data
Data available: Yes

Additional Information
NIH NIMH Data Archive (NIH NIDA) Repository Collection ID #2124
https://nda.nih.gov/edit_collection.html?id=2124
